# Variability of Intensive Care Admission Decisions for the Very Elderly

**DOI:** 10.1371/journal.pone.0034387

**Published:** 2012-04-11

**Authors:** Ariane Boumendil, Derek C. Angus, Anne-Laure Guitonneau, Anne-Marie Menn, Christine Ginsburg, Khalil Takun, Alain Davido, Rafik Masmoudi, Benoît Doumenc, Dominique Pateron, Maité Garrouste-Orgeas, Dominique Somme, Tabassome Simon, Philippe Aegerter, Bertrand Guidet, on behalf of the ICE-CUB study group

**Affiliations:** 1 Unité de Recherche en Épidémiologie Systèmes d'Information et Modélisation U707, Institut national de la santé et de la recherche médicale, Paris, France; 2 UMRS-707, Université Pierre et Marie Curie Univ Paris 06, Paris, France; 3 UPRES EA 2506 «Santé Environnement Vieillissement», Université de Versailles Saint-Quentin, Paris, France; 4 Department of Critical Care Medicine, University of Pittsburgh School of Medicine, Pittsburgh, Pennsylvania, United States of America; 5 Emergency Department Hôpital Bichat, Assistance Publique - Hôpitaux de Paris, Paris, France; 6 Emergency department Hôpital Victor Dupouy, Argenteuil, France; 7 Emergency Department Hôpital Cochin, Assistance Publique - Hôpitaux de Paris, Paris, France; 8 Emergency Department Hôpital Européen Georges Pompidou, Assistance Publique - Hôpitaux de Paris, Paris, France; 9 Emergency Department Hôpital Bicêtre, Assistance Publique - Hôpitaux de Paris, Paris, France; 10 Emergency Department Hôpital Saint-Antoine, Assistance Publique - Hôpitaux de Paris, Paris, France; 11 Medical-Surgical Intensive Care Unit, Saint Joseph Hospital Network, Paris, France; 12 Geriatric Unit Hôpital Européen Georges Pompidou, Assistance Publique - Hôpitaux de Paris, Paris, France; 13 Unité de Recherche Clinique Est Hôpital Saint-Antoine, Assistance Publique - Hôpitaux de Paris, Paris, France; 14 Université Pierre et Marie Curie Univ Paris 06, Department of Pharmacology, Paris, France; 15 Department of Public Health Hôpital Ambroise Paré, Assistance Publique - Hôpitaux de Paris, Boulogne-Billancourt, France; 16 Medical ICU Hôpital Saint-Antoine, Assistance Publique - Hôpitaux de Paris, Paris, France; University of Pittsburgh Medical Center, United States of America

## Abstract

Although increasing numbers of very elderly patients are requiring intensive care, few large sample studies have investigated ICU admission of very elderly patients. Data on pre triage by physicians from other specialities is limited. This observational cohort study aims at examining inter-hospital variability of ICU admission rates and its association with patients' outcomes. All patients over 80 years possibly qualifying for ICU admission who presented to the emergency departments (ED) of 15 hospitals in the Paris (France) area during a one-year period were prospectively included in the study. Main outcome measures were ICU eligibility, as assessed by the ED and ICU physicians; in-hospital mortality; and vital and functional status 6 months after the ED visit. 2646 patients (median age 86; interquartile range 83–91) were included in the study. 94% of participants completed follow-up (n = 2495). 12.4% (n = 329) of participants were deemed eligible for ICU admission by ED physicians and intensivists. The overall in-hospital and 6-month mortality rates were respectively 27.2% (n = 717) and 50.7% (n = 1264). At six months, 57.5% (n = 1433) of patients had died or had a functional deterioration. Rates of patients deemed eligible for ICU admission ranged from 5.6% to 38.8% across the participating centers, and this variability persisted after adjustment for patients' characteristics. Despite this variability, we found no association between level of ICU eligibility and either in-hospital death or six-month death or functional deterioration. In France, the likelihood that a very elderly person will be admitted to an ICU varies widely from one hospital to another. Influence of intensive care admission on patients' outcome remains unclear.

**Trial Registration:**

ClinicalTrials.gov NCT00912600

## Introduction

Admitting a very elderly patient to the ICU is one of the most difficult clinical challenges in medicine. The treating physician must quickly balance a complex set of acute signs and symptoms, patient and family preferences for life-sustaining therapies, estimates of likely survival and quality of life with and without intensive care, and a sense of stewardship of scarce expensive societal resources. Admitting a very elderly person to the ICU is sometimes seen as overly aggressive when prospects of long-term survival are dim, yet denial of ICU admission can incur accusations of unfair rationing [1], [2].

Unfortunately, there are few data to help guide clinicians in this area. Several studies suggest older age is associated with a decreased likelihood of ICU admission [3]–[10], but the decision process has received only limited study [3]–[6], [6], [10]–[18]. In France -as in many countries-, emergency departments are the main source of admission of very elderly patients to the ICU. Emergency rooms have a function of triage: the vast majority of acute patients are admitted to the hospital through the emergency room. Practically, the emergency physician suggests a potential case to the intensivist who in turn decides to accept it. Therefore final selection is a two-step process, each step having its own variability. Triage made by emergency physicians, however, has received little attention [10], [19] and has never been analysed through prospective large scale study. Moreover, estimates of the benefits of ICU admission, especially in the very elderly, are sparse, in part because of numerous methodologic and ethical challenges [13], [17], [20], [21]. As concerns mount about optimal use of intensive care, especially towards the end of life [22]–[24], more information on the whole ICU admission decision process and its consequences is needed.

The purpose of this study was to investigate variability of ICU admission rate of very elderly patients arriving in Emergency Departments (EDs) with conditions that potentially warrant ICU admission across multiple hospitals in the Paris metropolitan region. Specifically, we wished to understand whether ICU admission decisions were a function of local characteristics of individual hospitals, and whether differences in thresholds for ICU eligibility across hospitals were associated with differences in patient outcomes.

## Methods

### Participants

We designed the study, Intensive Care-Elderly CUB-Réa (ICECub), as a prospective multicenter observational cohort study of subjects arriving in the ED. Subjects had to be aged at least 80 years and be diagnosed by the ED physician with one of 74 conditions identified previously as potentially warranting ICU admission [19]. Briefly, these 74 conditions were identified by first taking all ICU admission criteria published by the Society of Critical Care Medicine [25], translating them into French, and then refining them to reflect local consensus via the Delphi method [19].

### Study design, procedures and variables

The study was conducted in 15 acute care hospitals in the Paris metropolitan region. Participating centers had a total number of 227 ICU beds, representing 22% of all ICU beds of the Paris metropolitan region. To minimize seasonal bias, each hospital enrolled patients for a 1-year period. The first site commenced in November 2004 and the last patient was enrolled in January 2006. Follow-up was completed in the fall of 2006, the clinical data were compiled, cleaned and locked in mid-2007, and analyses completed in 2008.

**Figure 1:**
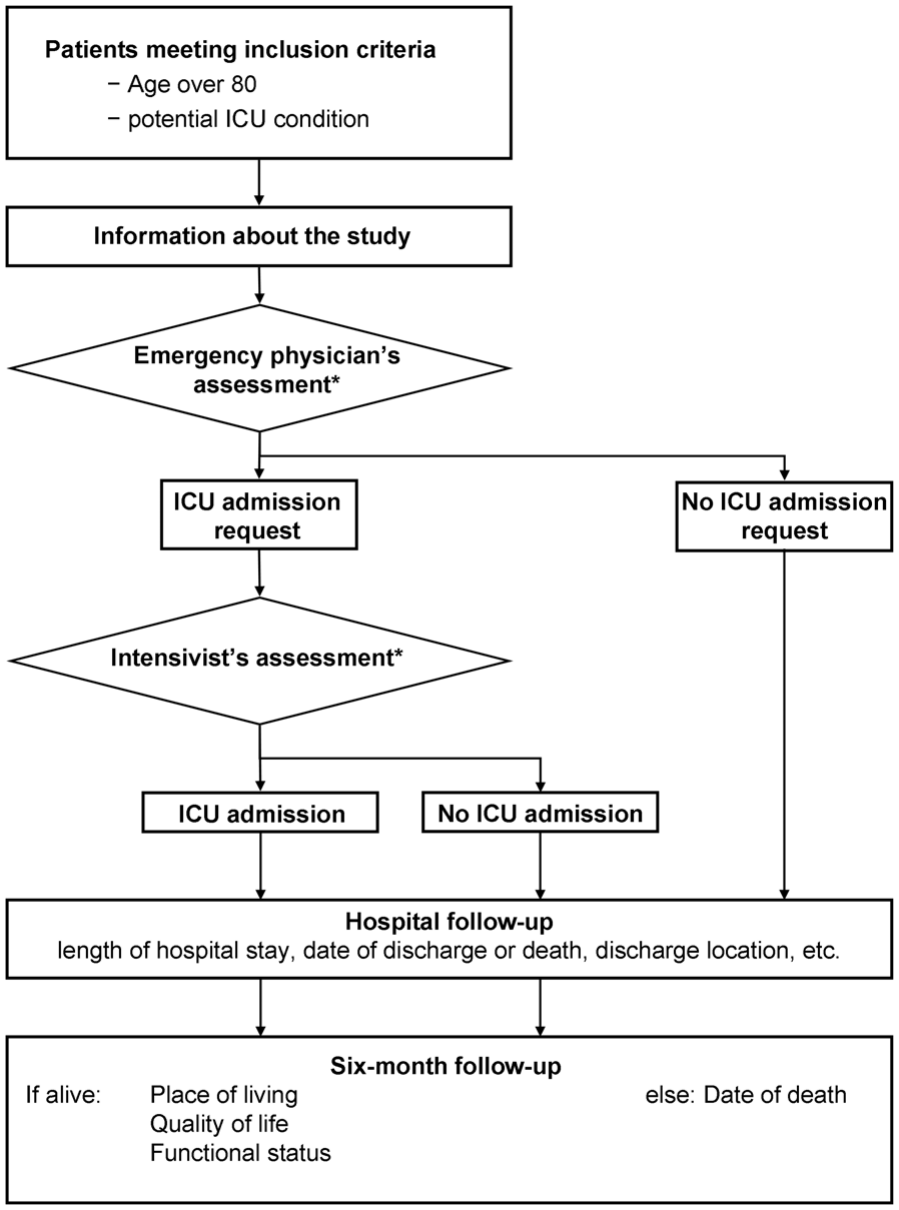
Inclusion and follow-up in the ICE-CUB study. *documented in the CRF, including complete evaluation of the patient's state: functional status, comorbidities, medication, falls, recent hospitalization.

Inclusion and follow-up of patients are described in figure 1. A case report form (CRF) was completed for each patient meeting the inclusion criteria. At inclusion, the ED physician evaluating the patient recorded age, gender, main diagnosis, chronic disease(s) (presence of chronic respiratory disease illness, chronic cardiac illness, chronic neurological illness, dementia and cancer as assessed by the evaluating physician), number and type of medications, recent hospitalizations, presence of dementia, functional status, gait and mobility, urinary and fecal continence, nutritional status (global appreciation), and whether decubitus ulcers were present. Severity of illness was measured with the Mortality Prediction Model_0_ (MPM_0_) [26]. Functional status was measured using the Katz activities of daily living (ADL) scale [27]. The emergency physician's assessment of the patient's need for ICU admission was also recorded. Patients were classified as deemed by the emergency physician to be i) eligible for ICU admission, ii) too sick to be admitted (no expected benefit from critical care treatment), or iii) too well to be admitted. The classification was left to the discretion of the physician, no specific guidelines were provided. If ICU admission was requested by the emergency physician, the intensivist's evaluation of the patient's need for ICU admission was recorded using the same classification. The final decision on ICU admission and characteristics of subsequent hospital stay were also recorded. Data were checked and completed by trained study coordinators at each site every week.

Study coordinators assessed the patients' functional status and quality of life 6 months after ED presentation via telephone interview. Dates of deaths occurring within the first 6 months following the ED visit were determined by study coordinators via follow-up phone calls to the patient's families, general practitioner, and relevant hospital as necessary.

### Hospital variables

The total number of ED visit and the number of ED visit of patients over 80 during the study period were extracted from hospital administrative databases. We also extracted information about each center's intensive care services from CUB-Réa, a database that has been collecting data from 36 ICUs in the Paris area since 1992 [28], [29]. Information included the total number of admissions, the observed to expected hospital mortality ratio (calculated using the simplified acute physiology score II [30]), the number of patients over 80, the number of beds, and the bed occupancy rates.

### Study outcomes

We focused on three outcome domains: physician assessments of ICU eligibility, in-hospital and 6-month mortality, and changes in functional status ; where a change in functional status was defined as a minimum of one point change in at least one dimension of the ADL with respect to baseline during the 6 months following the ED visit.

### Completeness

To evaluate how completely the study captured all potentially eligible subjects, we audited each site for one week randomly drawn from the inclusion period, excluding the first and last months. A study coordinator and a member of the steering committee reviewed the emergency department charts to estimate the number of patients missed during the randomly chosen week.

### Ethics

The study was approved by the Committee for Patient Protection (CPP), the Consultative Committee for Treatment of Health Research Information (CCTIRS), and the ethics committee of the French Society of Intensive Care. Because the study was observational, CPP and CCTIRS waived the need for written informed consent. As required by the French computer watchdog authority and CCTIRS, patients meeting the inclusion criteria (or their relatives) were informed of their inclusion, the follow-up process, and their right to consult their data, in a leaflet given to them in the ED.

### Statistical analyses

Univariate comparisons between eligible and non-eligible patients were based on a *t* test and the chi-square test for continuous and categorical variables. Relationships between continuous variables and the different outcomes were investigated. Collinearity of predictors was investigated using Hoeffding D [31].

Multilevel logistic regression analysis was used to investigate independent patient and center co-variables associated with physician assessments of ICU eligibility and with mortality at discharge and at 6 months. The variable “hospital” was used as a cluster variable. For each outcome we first estimated an ‘empty’ model which only included a random intercept and allowed us to detect a possible center effect. We then included patients' individual characteristics to investigate their role in inter-center differences. Finally, we added hospital variables to determine their influence on this “hospital effect”.

Two measures were used to estimate the hospital effect: the intraclass correlation coefficient (ICC) and the median odds ratio (MOR) [32]. As explained by Merlo et al. [33] “*Consider two persons with the same covariates, chosen randomly from two different clusters. The MOR is the median odds ratio between the person of higher propensity and the person of lower propensity.”*

The standardized eligibility rates per hospital are computed as the sum of predicted eligibility probability across one hospital divided by the number of patients actually considered eligible by both ED and ICU physicians.

All analyses were performed with *R* statistical software [34], SAS V8 (SAS Institute) and MLWin 2.02 (Multilevel Models Project, Institute of Education).

## Results

### Study population

Characteristics of the 15 participating centers are presented in table 1. Five hundred and seventy eight thousand patients visited the 15 EDs during the study period, among which 50,669 (8.8%) were aged over 80 years old. We enrolled 2,646 patients over 80 who presented to EDs with conditions that may have triggered an ICU admission. Sites contributed between 9 and 372 cases. Our random audit suggested that 62% of all potential ICU candidates over 80 were enrolled in the study (with a range of 36 to 88% of all potential ICU candidates over 80 across sites). Age was not statistically different in patients enrolled and not enrolled in the study. According to this result, 1 in 16 ED visitors over 80 years of age potentially required ICU admission, representing an average of 0.71 cases per ED per day.

**Table 1:** Hospital characteristics.

	Emergency Department		Intensive Care Unit variables										Specific ICE-CUB variables						
	*Visits*		*Characteristics*		*Stays*								*Patients*			*Triaging physicians*			
	*All patients*	*Patients over 80*			*All patients*				*Patients over 80*							*ED*		*ICU*	
Center	n	n	Beds n	Occupancy rate	n	Mean age	Hospital death rate	SMR*	n	Mean age	Hospital death rate	SMR*	Incl. n	ICU cand. n	Mean age	Mean age (n)	Female %	Mean age (n)	Female %
1	31 898	4 141	8	0.82	485	58	31	0.74	64	84	48	0.99	107	9	86	34 (107)	65	41 (19)	5
2	33 595	2 455	16	0.86	700	62	31	0.81	125	85	40	0.91	112	24	86	37 (112)	43	40 (44)	32
3	42 959	3 007	24	0.66	908	58	17	0.57	118	84	31	0.68	372	28	87	35 (367)	23	37 (32)	38
4	52 000	6 500	20	1.03	1057	57	21	0.79	134	84	27	0.82	353	40	87	35 (350)	76	32 (81)	21
5	65 496	4 585	24	0.88	1192	55	20	0.65	118	84	24	0.7	315	41	88	35 (315)	2	32 (90)	30
6	41 210	2 885	20	0.79	613	64	36	0.74	125	85	49	0.86	320	33	89	39 (319)	52	37 (54)	31
7	25 985	1 575	8	0.79	285	59	23	0.72	47	85	42	0.73	90	9	87	38 (90)	31	39 (23)	26
8	35 909	2 514	15	1.29	955	52	18	0.49	94	86	39	0.7	43	12	89	37 (41)	63	35 (13)	38
9	9 458	662	26	0.58	1145	58	23	0.77	168	85	21	0.87	9	4	87	32 (8)	33	34 (6)	67
10	39 000	4 832	10	0.86	554	55	17	0.49	58	84	33	0.82	63	13	87	41 (63)	90	41 (24)	33
11	47 941	3 356	10	0.86	572	58	23	0.73	74	85	26	0.46	82	23	87	35 (82)	48	34 (24)	32
12	45 811	4 800	14	0.86	958	59	23	0.76	180	85	29	0.87	134	52	87	37 (133)	58	40 (64)	3
13	27 418	3 797	10	1.11	213	63	26	0.92	41	84	27	0.8	198	11	88	42 (197)	83	32 (46)	6
14	39 368	2 756	10	0.71	332	55	19	0.79	19	83	37	1.21	98	10	89	36 (98)	61	35 (17)	47
15	40 052	2 804	12	0.86	550	59	27	0.81	74	83	38	0.99	350	20	87	34 (347)	77	44 (68)	3

* Standardized mortality ratio based on mortality predicted by SAPS II score.

Table 2 and 3 show the baseline characteristics of included patients. Ten percent of patients were at least 95 years old; 58.8% of patients were independent with respect to all 6 activities listed in Katz's ADL scale, and 14.4% were dependent for all activities. Four in 5 patients had a chronic illness, and 1 in 5 had dementia. There were few missing data: the rate ranged from 0.6% to 31.1%. Severity could be estimated in 94% of cases.

**Table 2:** General characteristics and potential ICU conditions of the patients according to physician decisions.

	all	Eligible for ICU admission	Non eligible		Missing values
N	2646	329	2317		
***General characteristics***					
Age (y)	87.41 (5.17; 86 ; 83–91)	85.3 (4.19 ; 84 ; 82–87)	87.7 (5.22 ; 87 ; 83–92)		
Women % (n)	62.6% (1658)	58.7% (193)	63.2% (1465)	*	
Place of residence					2.5% (66)
Home	78.5% (2024)	87.8% (281)	77.1% (1743)	*	
Nursing home	19.8% (510)	10.3% (33)	21.1% (477)		
Hospital	1.7% (46)	1.9% (6)	1.8% (40)		
Living alone	56.6% (1169)	55.1% (158)	56.8% (1011)		21.9% (580)
Accompanying relative in ED	41.3% (1093)	47.1% (154)	40.8% (939)	*	0.6% (15)
***Potential ICU conditions and comorbidities***					
Condition potentially warranting ICU admission according to main organ system				*	-
A – Cardiac	24.5% (647)	20.1% (67)	25.0% (580)		
B – Drugs (use and overdose)	1.9% (50)	2.4% (8)	1.8% (42)		
C – Endocrine	1.7% (46)	3.3% (11)	1.5% (35)		
D – Surgical	0.8% (22)	1.8% (6)	0.7% (16)		
E – Neurological	12.7% (335)	6.1% (20)	13.6% (315)		
F – Gastrointestinal	4.1% (109)	8.8% (29)	3.5% (80)		
G – Pulmonary	22.2% (588)	31.9% (105)	20.8% (483)		
H – Miscellaneous	7.3% (192)	11.8% (39)	6.6% (153)		
I – Laboratory values (newly discovered) and physical findings (acute onset)	18.1% (478)	7.6% (25)	19.5% (453)		
J – Other potential ICU admission diagnosis	6.8% (179)	5.8% (19)	6.9% (160)		
Chronic respiratory disease‡	18.9% (477)	24.9% (77)	18.1% (400)	*	4.7% (124)
Chronic cardiac illness‡	64.2% (1625)	60.2% (189)	64.7% (1436)		4.3% (114)
Chronic neurological illness‡	15% (379)	9.6% (30)	15.8% (349)	*	4.5% (118)
Cancer‡	10.5% (260)	8% (25)	10.8% (235)	*	6.4% (169)

Results for continuous and categorical variables are presented respectively as the mean (sd; median ; Inter-Quartile Range) and % (n).

* significant difference (P<0.05).

† assessed using Katz's Activities of Daily Living scale (ADL).

‡ as assessed by the evaluating physician.

**Table 3:** Geriatric conditions of the patients according to physician decisions.

	all	Eligible for ICU admission	Non eligible		Missing values
N	2646	329	2317		
***Geriatric conditions***					
Decubitus ulcer	5.8% (150)	4.4% (14)	6.0% (136)		2.6% (69)
Dementia‡	19% (480)	9.3% (29)	20.5% (451)	*	5.1% (134)
Medication #	5.46 (3.22; 5 ; 3–7)	5.35 (3.43 ; 5 ; 3–7)	5.48 (3.19 ; 5 ; 3–7)		11.8% (312)
Functional status assessed in the ED†	4.08 (2.18; 5 ; 2.5–6)	4.86 (1.70 ; 6 ; 4–6)	3.97 (2.22 ; 5 ; 2.5–6)	*	11.7% (309)
Nutritional status				*	3.3% (87)
normal appearance	65.5% (1675)	72.4% (231)	64.5% (1444)		
appears somewhat malnourished	19.6% (501)	18.8% (60)	19.7% (441)		
appears malnourished/emaciated	15% (383)	8.8% (28)	15.8% (355)		
Position				*	6.6% (175)
stable	49.4% (1221)	58.6% (181)	48.1% (1040)		
unstable	27.5% (679)	22.6% (70)	28.1% (609)		
impossible/confined in bed	23.1% (571)	18.7% (58)	23.7% (513)		
Recent hospitalization					
less than one month ago	22.8% (440)	19.9% (51)	23.4% (389)		
between one and six month(s) ago	21.4% (414)	18% (46)	22% (368)		
More than six months ago	55.7% (1076)	62.1% (159)	54.8% (917)		
Fall(s) during the previous 6 months					31.1% (822)
None	70.3% (1283)	72.9% (178)	69.9% (1105)		
One	13.6% (249)	14.3% (35)	13.5% (214)		
Between 1 and 3	8.7% (158)	7% (17)	8.9% (141)		
More than three	7.4% (134)	5.7% (14)	8% (120)		

Results for continuous and categorical variables are presented respectively as the mean (sd; median ; Inter-Quartile Range) and % (n).

* significant difference (P<0.05).

† assessed using Katz's Activities of Daily Living scale (ADL).

‡  as assessed by the evaluating physician.

### Triage decisions

**Figure 2:**
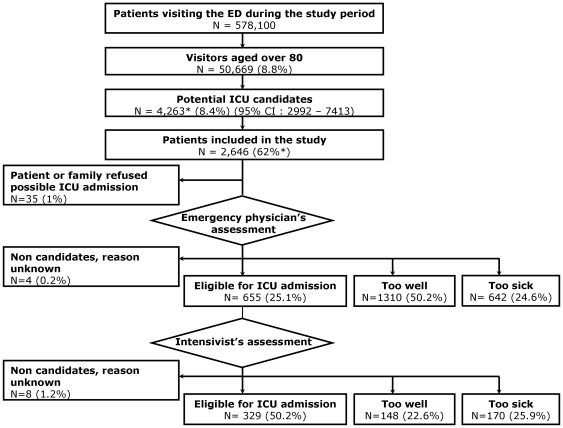
Flow Chart. * To evaluate exhaustiveness of patient inclusion in the study, one week was randomly drawn from the inclusion period, excluding the first and last month in each center. A study coordinator and a member of the steering committee reviewed the emergency department charts to estimate the number of patients missed during the randomly chosen week. Exhaustiveness was defined as the number of included patients divided by the total number of patients who should have been included in the study (sum of missed and included patients). It was extrapolated based on the estimation in each center: 62% (36%–88%).

figure 2 shows how the included patients were triaged. Thirty-five patients did not wish to receive intensive care. The ED physicians requested ICU admission for 25% of patients, and the ICU physicians refused 50% of these requests. Non eligible patients could be either admitted to intermediate care units or transferred to another hospital. A total of 329 patients were eligible for ICU admission according to both the emergency and ICU physicians (12.4%), while respectively 55.1% (n = 1458) and 30.7% (n = 812) of included potential ICU candidates were refused ICU admission because they were considered too well or too sick by emergency or ICU physicians.

Nine patients eligible for ICU admission were not admitted to an ICU, and 7 patients who were not considered eligible were finally admitted to an ICU. The final ICU admission rate was 12.4% (n = 327).

### Variability of physicians' assessment of ICU eligibility

Two centers underwent major changes in ED organization and stopped recruiting patients before the end of the study. The 52 patients enrolled by these centers were excluded from the analysis. Among the 2594 patients enrolled in the other 13 centers, patients who did not wish to receive intensive care were also excluded. The analysis thus focused on 2559 patients. Patients with missing values were also excluded from the analysis (see table 4).

**Table 4:** Multivariate models of outcome following the ED visit.

Outcome	ICU eligibility	In-hospital death	Death at 6 months	Death or functional deterioration at 6 months*
Number of observations used	1834	2095	1870	1870
	OR (95%CrI)	OR (95%CrI)	OR (95%CrI)	OR (95%CrI)
**Fixed effet**				
Age (grand mean centered) per year	0.91 (0.87–0.94)		1.04 (1.02–1.06)	1.05 (1.03–1.07)
ADL per one point increase	1.32 (1.19–1.46)	0.79 (0.75–0.84)	0.85 (0.80–0.91)	0.86 (0.81–0.90)
Demented (yes vs no)		0.61 (0.44–0.85)		
Cancer (yes vs no)	0.60 (0.33–1.05)		2.59 (1.74–3.90)	1.99 (1.38–2.97)
Normal appearance (vs appears emaciated)	0.42 (0.20–0.82)		0.82 (0.54–1.24)	0.96 (0.63–1.41)
Appears somewhat malnourished (vs appears emaciated)	1.06 (0.68–1.60)		0.48 (0.33–0.70)	0.57 (0.39–0.83)
Decubitus ulcer (yes vs no)		1.53 (0.97–2.26)		
Psychotropic drugs (yes vs no)	0.66 (0.45–0.95)			
Diuretics (yes vs no)			1.31 (1.05–1.63)	1.35 (1.11–1.65)
**Random effect**				
*Random intercept:*				
intercept variance (V(U0j))**	0.722 (0.412)	0.245 (0.154)	0.027 (0.033)	0.036 (0.041)
Residual intraclass correlation coefficient (ICC)	18%	6.93%	0.81%	1.08%
Median Odds Ratio***	2.25 (1.60–3.58)	1.60 (1.29–2.15)	1.17 (1.03–1.38)	1.20 (1.03–1.43)

Results are adjusted for severity (logit of the MPM_0_ score corrected from the points attributed to age) and main presenting problem as assessed in ED.

* defined as a one-point loss on at least one dimension of the ADL score six months after the ED visit;

** estimated true inter-hospital variance;

*** The Median Odds Ratio (MOR) is defined as the median value of the odds ratio between the hospital at highest risk and the hospital at lowest risk for two randomly chosen hospital.

Variables independently associated with ICU eligibility are described in table 4. Logit of the MPM_0_ score corrected from the points attributed to age was used to adjust for severity of the patient (OR for one point increase 1.77, 95% CI 1.51–2.08). Severity, main presenting problem, age, functional status, underlying cancer, nutritional status and psychotropic drug use explained about 28% of the variance in the proportion patients considered eligible for ICU admission. The model was well calibrated (H&L goodness-of-fit 9.6; p = 0.29−χ^2^_8ddl_).

The crude ICU eligibility rate ranged from 5.6% to 38.8% in the different participating centers. The empty multilevel logistic model of ICU eligibility gave an ICC of 16.5% and an MOR of 2.16 (95% CI, 1.58–3.46). The center effect did not seem to diminish after adjustment for individual patient characteristics (MOR 2.25, 1.60–3.58; hospital-related variance 18%). No hospital characteristic was associated with a decrease in inter hospital variability. ICU beds occupancy rates had no influence on ICU eligibility.

### Outcome: determinants and inter-hospital variability

The overall in-hospital mortality rate was 27.2%. The in-hospital mortality was 33% in ICU eligible patients whereas it was respectively 58% and 8% in patients considered too sick and too well (p-value of overall difference <0.0001). Only 151 patients were lost to follow-up. Excluding these patients, the overall 6-month mortality rate was 50.7%. Table 5 shows outcome according to the physician's assessment of the patient's need for ICU admission.

**Table 5:** ICU admission and outcomes according to physicians' decisions.

	all	Eligible for ICU admission	Non eligible		Missing values
N	2646	329	2317		
ICU admission	12.4% (327)	97.3% (320)	0.3% (7)	*	
Hospital death	27.2% (717)	32.8% (108)	26.3% (609)	*	
Probability of death †	31% (20%; 20% ; 16%–41%)	35% (22%; 29%; 16%–45%)	30% (19% ; 20% ; 17%–40%)	*	6.0% (159)
Death (in hospital + at 6 months)	50.7% (1264)	50.6% (157)	50.7% (1107)		5.7% (151)
Functional status at six months‡	4.26 (1.92; 5; 3–6)	4.71 (1.67; 5.5 ; 4–6)	4.19 (1.95; 5 ; 3–6)	*	11.8% (145)
Death or functional deterioration§	63.3% (1433)	63.7% (177)	63.2% (1256)		14.4% (382)

Results for continuous and categorical variables are presented respectively as the mean (sd; median ; Inter-Quartile Range) or % (n).

* significant difference (P<0.05).

† estimation based on the Mortality Probability Model 0 (MPM0).

‡ assessed using Katz's Activities of Daily Living scale (ADL).

§ defined as a one-point loss in at least one dimension of the ADL score six months after the ED visit.

Among the 1230 patients alive 6 months after their ED visit, 1085 had their functional status evaluated: 33.7% were independent for all activities listed in Katz's scale and 16.2% were unable to perform at least one activity they had been able to perform at the time of the ED visit. Six months after the ED visit, 57.5% of patients had died or experienced a functional deterioration.

Variables independently associated with 6-month outcomes are shown in table 4. Predictors of in-hospital death were mainly related to immediate severity (severity score, condition potentially warranting ICU admission, and decubitus ulcers), whereas predictors of 6-month outcomes were mainly related to general health status (nutritional status, underlying disease and diuretic prescription). Functional status was a major predictor of short-term and mid-term outcome.

The in-hospital mortality rate varied across the participating centers, as did the 6-month mortality rate and the rate of death and functional deterioration at 6 months: the respective ICCs were 9.5%, 5.5% and 4.1%, and the respective MORs (95%CI) were 1.75 (1.41–2.39), 1.52 (1.27–1.93) and 1.43 (1.21–1.78).

Patient characteristics appeared to explain a large part of the residual variance in outcome, especially at 6-month (see models in table 4).

### Association of ICU use and outcome

**Figure 3:**
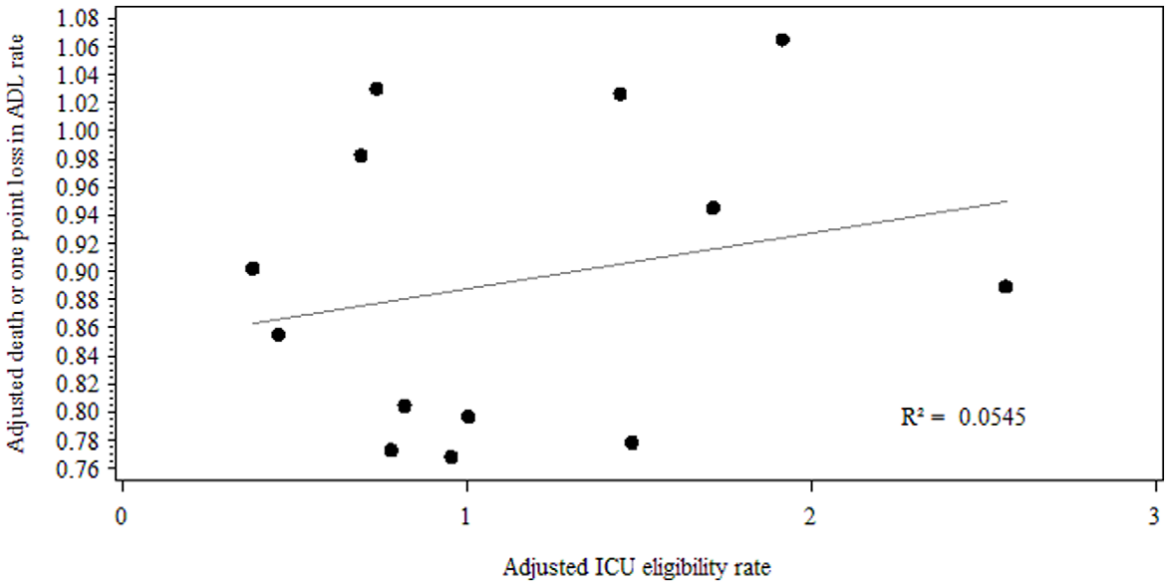
Association between ICU admission rate and six-month outcome. Adjusted 6-month mortality or one-point loss of ADL rate versus adjusted ICU eligibility rate.

**Figure 4:**
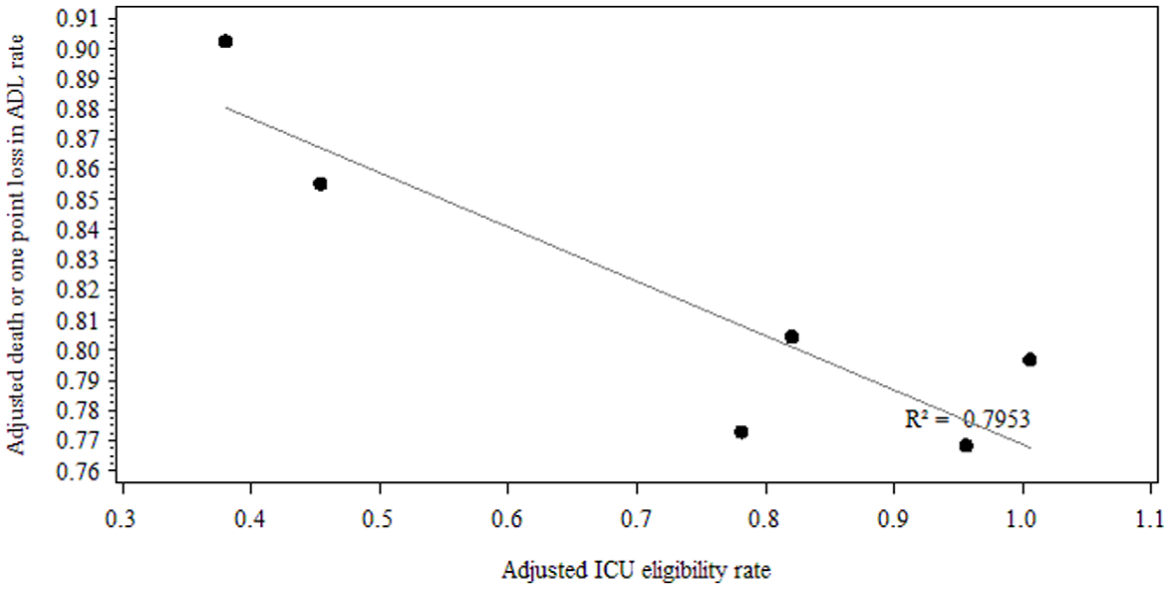
Association between ICU admission rate and six-month outcome among large hospitals (over 150 inclusions). Adjusted 6-month mortality or one-point loss of ADL rate versus adjusted ICU eligibility rate.

In order to investigate association between level of admission of patients over 80 and 6-month outcome, we plotted the adjusted outcome rates against the adjusted ICU admission rates. Results for long-term outcome are shown in figure 3. Estimates of association of standardized eligibility rates and outcomes are shown in table 6. A non significant relationship was found between the ICU eligibility rate and outcomes. However, we found a negative correlation between adjusted ICU eligibility rates and all three outcomes in large centers (over 150 inclusions) (figure 4): the more ICUs are likely to admit old patients, the best the short and long-term result of hospitalization for all elderly potentially requiring ICU admission.

**Table 6:** Association of standardized ICU eligibility rate with all standardized outcomes.

	Standardized in-hospital mortality	Standardized 6-month mortality	Standardized rate of 6 month death or one point loss in ADL	Standardized rate of 6 month death or one point loss in ADL (in large centers - over 150 inclusions)
R^2^	0.008	0.213	0.054	0.795
p-value (Pearson correlation test)	0.767	0.113	0.443	0.017

## Discussion

This is the first prospective multicenter study that describes and assesses the whole ICU triage process of old patients from the Emergency Department to the ICU.

There were several findings from this study. First, French hospitals are faced very frequently with potential ICU admission decisions for the very elderly. Second, only 1 in 8 elderly patients with potential criteria for ICU admission are actually admitted, which is a much lower rate than that reported previously, perhaps because other studies failed to examine the ED physician decision-making and therefore underestimated the size of the denominator population [3]–[6], [11]–[15], [35]. For example, in a recent study of all patients over 18 with a request for ICU admission performed in 11 European ICU, Sprung et *al.* [17] showed an unadjusted refusal rate of 23% in patients aged between 75 and 84 years old and of 36% in patients aged over 85.

Third, ED and ICU physicians selected principally on the severity of acute physiologic derangment and relative lack of comorbidity, especially dementia, and functional impairment. Like Garrouste et *al.* and Rogriguez-Molina et *al.* [10], [15], we found that age and functional status prior to the ED visit were major determinants of ICU admission. In so doing, the physicians identified broadly a group of patients whose outcome, both in terms of mortality and functional status, fell in the middle range among all potential candidates. Of note, the 6 month outcomes for those who were admitted to the ICU was somewhat similar to that reported in other series [15], [36]–[41]. However, this observation does not mean physicians chose the ‘best’ candidates on average, nor does it mean that they made good individual decisions. The 2005 INSEE (Institut National de la Statistiques et des Etudes Economiques) age- and sex-standardized 6-month mortality in the general French population was 9%, two-thirds lower than that incurred by those patients considered ‘too healthy’ for ICU admission.

Fourth, although there was an ‘average’ physician behavior, that average was very different across centers, with some centers admitting 1 in 3 vs. others admitting 1 in 18. This several-fold difference was not explained by differences in patient characteristics. Variability in ICU admission decision in old patients has never been studied in depth. Sprung et *al*. showed a high variability in ICU triage decisions in the 11 European ICUS that participated in their study, not explained by patient severity [17]. The variability found in their study however might be explained by variation in hospital organization across countries. Unwanted variation in the use of healthcare technology is well-described, and the focus of much current attention, in US healthcare delivery, but is often assumed to be dependent in large part on economic incentives. It is therefore disappointing to see such large variation within a single payor government-run healthcare system. Furthermore, we were unable to determine any obvious hospital characteristics that would explain this variation suggesting that the variability is mainly related to physicians' beliefs.

Finally, because there was such variation in the use of ICU admission, we wondered whether hospitals with greater use would gain better patient outcomes. However, we were unable to show that greater use of ICUs, after adjusting for severity of illness, had any obvious impact on either short or long-term mortality. The negative relationship between rate of ICU admission and rate of death or functional deterioration six month after ED visit found in the 6 major centers should be interpreted with caution. Few papers addressed the benefit of ICU admission in old patients [13], [17], [20], [21]. Recently Sprung et *al*. [17] have shown a benefit of ICU admission in patients over 65. However, they only studied triage performed by intensivists. Moreover, they studied short term outcome defined as mortality at 28 days, such a close horizon impede any long term conclusion. Indeed in a recent work our team has shown that adjusted survival estimates sharply decreased after 30 days in admitted patients [42].

### Limitations

First, it was an observational study with no opportunity to leverage randomization to make inferences of causality. However, this approach yielded useful information on the use of intensive care for the elderly in routine practice. Second, the study was limited to hospitals in the Paris area of France, and our conclusions may not apply to other healthcare systems. Third, the lack of a significant association between adjusted outcomes and ICU admission rates may be due to a lack of statistical power, as the regression analysis included only the 13 centers for which exhaustive data were available.

### Conclusion

We found that the odds of ICU admission among very elderly patients presenting to an emergency department varied widely from one hospital to another. However, ICU admission did not appear to influence short-term or mid-term vital or functional outcome. Factors associated with long-term outcome identified in this study might help physicians in making their decision or designing further studies. We believe the use of these factors to make ICU admission decision in the ED will help reduce variation in ICU use and improve outcome of hospitalization. A prospective interventional study is ongoing aiming to address impact on mid-term mortality of guidelines for ICU admission of elderly patients arriving in Emergency Departments.
